# Initial experience of video endoscopic inguinal Lymphadenectomy in a center located at northeast brazilian region

**DOI:** 10.1590/S1677-5538.IBJU.2018.0521

**Published:** 2019-04-01

**Authors:** Aurus Dourado Meneses, Pablo Aloisio Lima Mattos, Walberto Monteiro Neiva Eulálio, Taíla Sousa de Moura Fé, Rodolfo Myronn de Melo Rodrigues, Marcos Tobias-Machado

**Affiliations:** 1 Divisão de Urologia, Hospital São Marcos, Teresina, PI, Brasil;; 2 Universidade Federal do Piaui, Teresina, PI, Brasil;; 3 Centro Universitário Uninovafapi, Teresina, PI, Brasil;; 4 Departamento de Urologia, Faculdade de Medicina do ABC, Santo André, SP, Brasil

**Keywords:** Penile Neoplasms, Lymph Node Excision, Minimally Invasive Surgical Procedures

## Abstract

**Introduction:**

Video endoscopic inguinal lymphadenectomy – VEIL – has emerged as an alternative to reduce post-surgical complications (PSC) in patients with penile cancer submitted to inguinal lymphadenectomy (IL). In some series, these PSC are observed in more than 50% of patients. The objectives of the present study are to describe the initial experience of VEIL in a Hospital in Teresina, PI, Brazil, and to analyze PSC incidence.

**Material and Methods:**

Retrospective descriptive study of patients submitted to VEIL from March 2014 to November 2015. Data were collected regarding surgical time, bleeding, complications, lymph node number, conversion, global complications, drainage time, cellulitis, lymphocele, cutaneous necrosis, miocutaneous necrosis and hospitalization time.

**Results:**

20 lower limbs of 11 patients were operated. Mean age was 51.4 (24-72) years. Mean surgical time was 85 (60-120) minutes. No patient showed intrasurgical complications, bleeding > 50 mL or conversion. Three surgeries evolved with lower limb edema, 2 with lymphoceles and one patient had cutaneous necrosis and another bulging of surgical wound. Mean time of hospitalization was 4 (2-11) days. A mean of 5.8 (1-12) lymph nodes were dissected in each surgery.

**Conclusion:**

VEIL is a safe and easy technique with lower incidence of PSC that can be reproduced in small centers.

## INTRODUCTION

The main site of metastasis of penile cancer is inguinal lymph nodes. Around 30% of patients show inguinal involvement at diagnosis, and this is a determinant factor of mortality related to this type of tumor (1, 2). Open inguinal lymphadenectomy (IL) is considered the gold standard of care for lymph node metastasis of penile cancer (3). However, this procedure has a high morbidity, including skin necrosis or post-operatory infection of surgical wound. Also, depending on the extension of lymphadenectomy, a higher frequency of lower limb edema, lymphocele, lymphedema and lymphorrea is observed (4).

In 2006, Tobias-Machado et al. described the Video Endoscopic Inguinal Lymphadenectomy – VEIL, in order to reduce morbidity, but maintaining the same oncologic principles of conventional technique (5). Since then, several series were published with good results. VEIL is easy and safe to perform in certain patients, but, although with promising initial results, this surgery is still not enough widespread and currently is being more performed in referral centers.

The objectives of the present study are to describe the initial experience with VEIL in a tertiary center in the northeast region of Brazil, to analyze the incidence of post-surgical complications, to critically analyze the technique and to verify its reproducibility in a median size hospital.

## MATERIALS AND METHODS

This is a retrospective and descriptive study, approved by the Ethical and Research Committee of the Institution under the number 61046316.6.0000.5584. Data were exclusively obtained in the charts. The study was funded only by the authors.

The study was performed at the Hospital São Marcos, in Teresina, PI, Brazil. Patients were operated from March 2014 to November 2015. Lymphadenectomy was performed in men with epidermoid carcinoma submitted to previous penis amputation and with high risk of lymph node metastasis. Surgery was performed by three experienced urologists and after at least an interval of four weeks following amputation. Indication criteria included: T2 and/or G2 tumors and/or with vascular or perineural invasion. No patient showed peripheral lymphadenopathy.

Patients were operated according to Tobias-Machado technique: 1) positioning of lower limb abducted and extended; 2) introduction of 3 ports in the femoral triangle; 3) obtaining a work space with gas; 4) separation of skin using electric scalp; 5) identification and dissection of saphenous vein magna up to oval fossa; 6) identification of femoral artery; 7) distal ligature of lymph node block on the vertex of the femoral triangle; 8) release of lymph node tissue until the great vessels above femoral surface; 9) distal ligature of saphenous vein magna; 10) control of saphenous-femoral junction; 11) final release of surgical specimen and field hemostasis; 12) removal through initial orifice; 13) vacuum drainage and closure of incisions.

Demographic data were evaluated and summarized in tables. Clinical data were evaluated and summarized in tables. The following peri-operatory data were analyzed: surgical time, bleeding, complications, lymph node count, and conversion to open surgery. Also, post-surgical data were also analyzed: global complications, drainage time, cellulitis, lymphoceles, cutaneous necrosis, miocutaneous necrosis and time of hospitalization. Intra-operatory bleeding was estimated according to the aspirated volume during the procedure.

Data were analyzed at R-Project. In order to compare means and proportions of the selected literature results with those of the present study, it was used Analysis of Variance (ANOVA) with Tukey post-test. Normality assumption was verified by the Shapiro-Wilk test, and all variables were approximately normal. Significance level was 5% (p-value<0.05) (6).

## RESULTS

Main demographic and clinical and pathological characteristics of patients are summarized in Table-1. Mean age was 51.4 (24-72) years. Eleven patients were operated: 6 were submitted to bilateral VEIL (non-simultaneous), 3 to simultaneous bilateral VEIL, and 2 unilateral VEIL with open IL in the contralateral limb, totalizing 20 surgeries. Three patients (27.2%) showed compromised lymph nodes and in 8 (72.8%) no metastasis was identified. Positive lymph node rate was 8.3%. Mean follow-up time was 111 (2-180) weeks. One patient died due to pulmonary metastatic disease 22 weeks after surgery and another 83 weeks after surgery, due to local recurrence. Two patients were lost to follow-up, and the remaining are alive, without signs of active disease, with periodic consultations.

**Table 1:** Demographic, clinical and pathological characteristics of patients.

Number	1	2	3	4	5	6	7	8	9	10	11
Age at diagnosis	46	64	44	60	55	55	72	24	38	46	62
Histologic type	epidermoid	epidermoid	epidermoid	epidermoid	epidermoid	epidermoid	epidermoid	epidermoid	epidermoid	epidermoid	epidermoid
Pathologic staging	T2	T2	T2	T3	T2	T3	T1	T2	T2	T2	T3
	N0	N0	N0	N0	N0	N0	N0	N1	N1	N0	N1
	M0	M0	M0	M0	M0	M0	M0	M0	M0	M0	M0
	G1	G2	G1	G3	G3	G1	G3	G2	G1	G1	G3
Technique	SBV	NSBV	OU + UV	NSBV	NSBV	NSBV	NSBV	NSBV	SBV	SBV	OU + UV
Number of positive lymph nodes	0	0	0	0	0	0	0	2	1	0	6
Follow up (weeks)	83	136	135	23	111	180	2	117	59	62	148
Current status	D	A	A	D	A	A	L	A	L	A	A

**SBV =** simultaneous bilateral **VEIL; NSBV =** non-simultaneous bilateral **VEIL; OU =** open unilateral; **UV =** unilateral VEIL; **D =** death; **A =** alive; **L =** loss of follow-up

Main surgical results are summarized in Table-2. Mean operatory time was 85 (60-120) minutes. A mean of 5.8 (1-12) lymph nodes were dissected in each surgery. Intra-operatory bleeding of all procedures was lower than 50 mL, therefore considered negligible. No patient presented intra-operatory complication or conversion to open surgery.

**Table 2:** Results of Analysis of Variance (ANOVA) to compare means and proportion in reference to the data of current study.

Variables	Current study	Romanelli P (6)	Pahwa HS (7)	Chaudhari R (12)	Kumar V (13)	Yuan JB (14)
Number of patients	11	20	10	14	20	12
Surgical mean time	85	119	144	194	97	92
P-value	Ref.	**0.038** *	**0.023** *	**0.015** *	0.442	0.64
Skin complications (%)	10	6	0	0	6	4.2
P-value	Ref.	0.605	**<0.001** *	**<0.001** *	0.112	**0.0321** *
Lymphatic complications (%)	25	27.2	20	27.2	30	12.5
P-value	Ref.	0.713	0.345	0.118	0.067	**0.039** *
Intrasurgical complications	0	0	0	0	0	0
P-value	Ref.	–	–	–	–	–
Mean number of dissected lymph nodes	5.7	8	10.6	7.6	9.3	10.5
P-value	Ref.	0.456	**0.0267** *	0.059	**0.038** *	**0.0302** *
Recurrence	2 in a mean follow-up of 28 months	2 in a mean follow up of 20 months	not discussed	0 in a mean follow-up of 48 months	0 in a mean follow-up of 16 months	not discussed
P-value	Ref.	0.901	–	0.623	0.0781	–

* Significant. Ref.: Reference for the test.

P-value: Error probability

Source: Author

**Figure-1:**
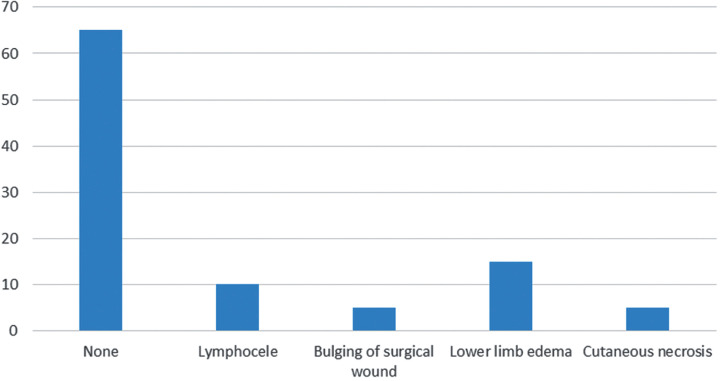
Post-surgical complications following VEIL.

Main post-surgical complications are described in Figure-1. Mean hospitalization time was 4 (2–11) days. One patient showed cutaneous necrosis of surgical wound and needed antibiotics. Mean drainage time was 8 days; some patients were discharged with drain in position due to copious secretion. The drain was removed during post-surgical ambulatory follow-up.

## DISCUSSION

Squamous cell penile carcinoma is a rare disease, especially in developed countries. Bad hygiene and poor health access are important risk factors for the high incidence and prevalence of this disease in under-developed regions. The present study was performed in a Hospital that attends disadvantaged patients and the present series included only men with low income and low education level. More often it manifests in middle aged men and forward, and in this series the epidemiologic data are coincident with those of literature (7).

In this series, the only histologic type was epidermoid carcinoma, and the literature appoints it as the most frequent subtype. Most patients presented pT2 stage tumors, which was the pathologic criteria for indication of lymphadenectomy for the included patients. This fact reflects that, even though this is an easily noted disease with a relative simple diagnosis, usually patients take a long time to seek medical help, increasing significantly morbidity of the disease and its treatments.

Since lymph node involvement is an important determining factor for mortality of this kind of tumor, IL must be performed even prophylactically. However, there are post-surgical complications, such as lymphedema, lymphocele, skin necrosis, deep venous thrombosis and thrombophlebitis, in 30 to 70% of patients submitted to IL (1, 2). VEIL main advantage in relation to IL is the reduction of morbidity. It is lower especially when related to skin (necrosis or post-surgical infection of surgical wound), and in several series these complications were not noted (8–11). General morbidity of VEIL varied from 20 (9, 10) to 33.2% (8), and lymphocele was the most frequent complication. Although VEIL has a significant morbidity rate, it is much lower than conventional IL. Pahwa et al., in a series of 10 patients, describes the incidence of infra-umbilical emphysema, that self-healed in 100% of patients. This was not observed in our patients (9). In that work, the incidence of positive lymph nodes was irrelevant, although the IL indication criteria were precise.

In this series of 20 VEIL, trans-surgical procedure was satisfactory in general, as the data presented in Table-2. The surgical time was excellent, statistically significant when compared to other series in literature (8, 9, 12), probably due to rapid proficiency gathered by the surgeons, that previously attended practical and theoretic courses, under the guidance of an experienced tutor. It seems that these specific VEIL courses are able to allow the reproducibility of the surgery with adequate safety.

Tobias-Machado (10) describes the mean surgical time of VEIL of 126 minutes versus 92 minutes of the conventional technique (11). However, it must be taken into account the learning curve of laparoscopy. In spite of the longer surgical time, the studies usually do not describe intra-operatory complications or conversions, and the longer surgical time is not a limiting factor for its use. There is a significant statistical difference in relation to the number of dissected lymph nodes, and in this series was lower than others in literature (9, 13, 14). This fact may be explained by the initial experience of the urologic department of our institution and with more procedures being performed, the results will equal to those of literature. A better understanding of superficial inguinal lymph node distribution will also contribute to a better lymph node dissection (15). Negligible bleeding and the lack of complications or conversions were also positive aspects of this initial series, reinforcing the safety of the procedure. Also, due the small size of the incision, VEIL has better cosmetic results. Therefore, patients are well satisfied with the results of the surgery, due to better cosmetics associated to lower morbidity (14).

Main complications of this series are summarized in Figure-1. Most frequently they were related to lymphatic drainage, including lower limb edema and lymphocele, minor complications that were conservatively treated. There was a significant statistical and unfavorable difference in relation to literature (14). The same was observed with other minor complications such as skin necrosis and bulging of surgical wound (9, 12, 14). We believe that all are related to the initial experience; therefore, proficiency is still not reached. With the inclusion of new patients, there will be a significant reduction of complications, approaching the results to the favorable number of literature.

Until nowadays, the bigger Latin-American study in literature included 20 patients submitted to 33 VEILS. 55% of patients were N0 and 45% N+. VEIL was performed bilaterally in 13 patients and 7 received unilateral VEIL associated to contra-lateral conventional IL. Mean surgical time was 119 minutes and the mean number of dissected lymph nodes was 8 by procedure. Global complication rate was 33.2%, and 27.2% were lymphatic. No patient showed cutaneous necrosis. In 6 patients whose internal saphenous vein was preserved, there was no lymphatic complications. Global survival time was 80% and cancer-specific survival was 90%, with a median follow-up of 20 months (8).

Recently, a European study compared the evolution of 33 VEIL versus 35 IL in 42 patients. Wound surgical complication rate was 6% in VEIL versus 68% in IL (p=0.0001). Also, patients submitted to VEIL had lower hospitalization time (4.9 days, p=0.0001). Other important aspects are the number of dissected lymph nodes and the number of positive lymph nodes, that was equal in both groups or slightly better in VEIL, that provided similar results but with a lower rate of post-surgical complications. However, in that study, as in others, the follow-up time was not long enough to propose an appropriate analysis of survival (13). The comparison of our results to other series is presented in Table-2.

Usually, patients die 2 years after diagnosis of primary lesion when not treated, due to major local and regional complications or development of metastasis in other organs (1). In our series, follow-up time was not ideal, mainly due to the low social and economic status of patients, that compromise awareness and commitment to treatment.

Although more than a decade has passed since the initial description of VEIL, there is still no study showing the applicability of this technique in small and medium size hospitals, therefore limiting its use to referral centers. The low number of patients, the retrospective aspect and the small follow-up time of our study precludes a more profound analysis of the oncologic safety of this technique. However, it was possible to demonstrate its use in small centers. Due to low incidence, multicenter studies are warranted to define the role of VEIL in the treatment of penile cancer, its oncologic safety and the potential use in other types of tumors.

## CONCLUSIONS

The study presents a reproducible and easy surgery, however many patients submitted to VEIL in this series presented some kind of morbidity, although minor, especially related to lymphatic drainage. The number of dissected lymph nodes was lower than in other important literature series, probably due to the ongoing learning curve. VEIL seems to be an alternative to reduce post-surgical morbidity of IL, easy to perform and reproducible in median size centers. However, randomized prospective studies are necessary to prove superiority of VEIL and to define its role in the treatment of penile cancer.
